# Oral ribose supplementation in dystroglycanopathy: A single case study

**DOI:** 10.1002/jmd2.12394

**Published:** 2024-03-04

**Authors:** R. M. J. Thewissen, M. A. Post, D. M. Maas, R. Veizaj, I. Wagenaar, M. Alsady, J. Kools, K. Bouman, H. Zweers, P. G. Meregalli, A. J. van der Kooi, P. A. van Doorn, J. T. Groothuis, D. J. Lefeber, N. C. Voermans

**Affiliations:** ^1^ Department of Neurology Donders Institute for Brain, Cognition and Behavior, Radboud University Medical Center Nijmegen The Netherlands; ^2^ Department of Rehabilitation Donders Institute for Brain, Cognition and Behavior, Radboud University Medical Center Nijmegen The Netherlands; ^3^ Translational Metabolic Laboratory, Department of Laboratory Medicine Radboud University Medical Center Nijmegen The Netherlands; ^4^ Department of Pediatric Neurology Donders Institute for Brain, Cognition and Behavior, Amalia Children's Hospital, Radboud University Medical Center Nijmegen The Netherlands; ^5^ Department of Gastroenterology Radboud University Medical Center Nijmegen The Netherlands; ^6^ Department of Cardiology Amsterdam UMC Amsterdam The Netherlands; ^7^ Department of Neurology Amsterdam UMC Amsterdam The Netherlands; ^8^ Department of Neurology Erasmus MC Rotterdam The Netherlands

**Keywords:** alpha‐dystroglycan, CDP‐ribitol, FKRP, muscular dystrophy, ribose, trial

## Abstract

Three forms of muscular dystrophy‐dystroglycanopathies are linked to the ribitol pathway. These include mutations in the isoprenoid synthase domain‐containing protein (*ISPD*), fukutin‐related protein (*FKRP*), and fukutin (*FKTN*) genes. The aforementioned enzymes are required for generation of the ribitol phosphate linkage in the O‐glycan of alpha‐dystroglycan. Mild cases of dystroglycanopathy present with slowly progressive muscle weakness, while in severe cases the eyes and brain are also involved. Previous research showed that ribose increased the intracellular concentrations of cytidine diphosphate‐ribitol (CDP‐ribitol) and had a therapeutic effect. Here, we report the safety and effects of oral ribose supplementation during 6 months in a patient with limb girdle muscular dystrophy type 2I (LGMD2I) due to a homozygous *FKRP* mutation. Ribose was well tolerated in doses of 9 g or 18 g/day. Supplementation with 18 g of ribose resulted in a decrease of creatine kinase levels of 70%. Moreover, metabolomics showed a significant increase in CDP‐ribitol levels with 18 g of ribose supplementation (*p* < 0.001). Although objective improvement in clinical and patient‐reported outcome measures was not observed, the patient reported subjective improvement of muscle strength, fatigue, and pain. This case study indicates that ribose supplementation in patients with dystroglycanopathy is safe and highlights the importance for future studies regarding its potential effects.

**Synopsis**

Supplementing oral ribose to a patient with limb girdle muscular dystrophy type 2I (LGMD2I) due to a homozygous *FKRP* mutation was well tolerated and resulted in a decrease of creatine kinase levels and subjective improvements of muscle strength, fatigue and pain.

## 1 INTRODUCTION

**FIGURE 1:**
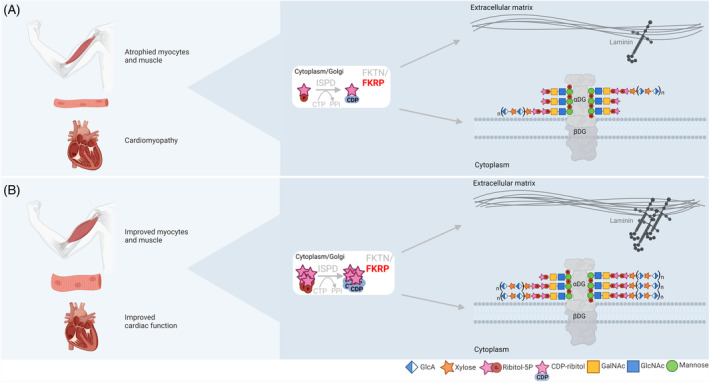
α‐Dystroglycan (DG) glycosylation and extracellular matrix (ECM) binding. (A) In patients with a *FKRP* mutation, α‐dystroglycan is hypoglycosylated resulting in, among others, loss of binding to the ECM causing muscular dystrophy. Some symptoms that these patients present with are limb girdle muscular dystrophy type 2I and cardiomyopathy. In the cytoplasm isoprenoid synthase domain‐containing protein (ISPD) produces cytidine 5′‐diphosphate (CDP)‐ribitol. This in turn can be used by fukutin (FKTN) and fukutin‐related protein (FKRP) in the Golgi apparatus to elongate the O‐glycan with two ribitol‐5‐phosphate residues. After a β1,2‐xylose (Xyl) and a β1,4 glucuronic acid (GlcA) is added to allow the α1,3‐Xyl‐β1,3‐GlcA repeats to finalize its glycosylation, glycosylated α‐DG will bind to laminin α‐2 in the muscle sarcolemma as well as to other components of the ECM. (B) Hypothesis for ribose supplementation. When extracellular ribose is transported into the cell it can be converted to ribitol‐5‐phosphate via reduction and phosphorylation. Having an excess of ribose could result in an higher production of CDP‐ribitol by the myocytes which could compensate for the reduced function of FKRP. This in turn could improve the patients' (cardiac) muscle strength via restoring glycosylation of αDG and thus extra anchor points for laminin to improve the binding between ECM and muscle cell membrane.

Primary dystroglycanopathies are caused by homozygous mutations in the *DAG1* gene, while secondary dystroglycanopathies are caused by mutations in genes involved in the alpha‐dystroglycan (αDG) glycosylation pathway. Mutations in the genes coding for isoprenoid synthase domain‐containing protein (*ISPD*), fukutin‐related protein (*FKRP*), and fukutin (*FKTN*) cause distinct forms of secondary dystroglycanopathies, ranging from the milder limb girdle muscular dystrophy (LGMD) to the severe multisystem Walker‐Warburg syndrome.^1^ So far, 18 genes have been identified as causative for dystroglycanopathies.^2^ Proper synthesis of O‐mannose glycans of αDG requires all proteins translated from these genes. αDG is part of the dystrophin‐glycoprotein complex (DGC) and a O‐mannose glycan acts as an anchor between laminin in the extracellular matrix (ECM) and the muscle cell membrane (Figure 1).^1^ Correct glycosylation of αDG is essential for muscle strength along with muscle function. Deficient glycosylation results in muscular dystrophy (Figure 1A).^1^, ^3^, ^4^ The ISPD enzyme produces the sugar cytidine diphosphate‐ribitol (CDP‐ribitol), which in turn is used in tandem by FKTN and FKRP. Both FKTN and FKRP transfer ribitol phosphate groups to one of the *O*‐mannose glycans of αDG.^5^, ^6^

Recent approaches in treatment of dystroglycanopathy focused on gene therapy, cell therapy, antisense oligonucleotide therapy, enzyme replacement therapy, corticosteroids, and small molecules.^1^, ^7^, ^8^ However, no causative treatment is yet available for ISPD, FKTN, and FKRP‐related dystroglycanopathies. Recent studies showed that supplementing *ISPD* deficient skin fibroblasts with ribitol and ribose increased concentrations of CDP‐ribitol and restored glycosylation of αDG.^5^, ^9^ Furthermore, ribitol feeding in *FKRP*‐mutant mice showed restoration of glycosylation.^10^ As a result, this enhanced laminin binding to dystroglycan, suggesting the potential for ribitol to enhance muscle function in dystroglycanopathy patients with an *FKRP* mutation. Additionally, a recent study developed a prodrug strategy with CDP‐ribitol derivates which successfully rescued αDG in *ISPD* deficient patient fibroblasts.^8^ However, ribitol still requires clinical trials to assess long‐term safety.^9^ Ribitol is formed by the reduction of its precursor ribose, which is a safe and commercially available food supplement.^11^, ^12^, ^13^ Patients with dystroglycanopathy could possibly benefit from ribose treatment for improved muscle function by increasing intracellular levels of CDP‐ribitol (Figure 1B).

This study aimed to determine if ribose is well tolerated in a patient with dystroglycanopathy due to an *FKRP* mutation as well as to study the effect of ribose supplementation on muscle strength and function in this patient.

## 2 METHODS

### 2.1 Study population

This case study recruited patients with a confirmed mutation in *ISPD*, *FKRP*, or *FKTN*. Participants were recruited via the Dutch patient association for patients with muscular diseases and by contacting patients personally.

Inclusion criteria were: (1) clinical presentation of isolated LGMD; (2) confirmed genetic diagnosis with mutations in *ISPD*, *FKRP*, or *FKTN*; (3) dysfunction of O‐glycosylation in muscle; and (4) normoglycemia before start of supplementation; (5) ≥18 years; and (6) able to give informed consent. Exclusion criteria were a previous severe hypoglycemia in the fed‐state or diabetes mellitus. The stop criterion was inadequate compliance to supplementation protocols.

### 2.2 Ethical considerations

This study with number NL58620.091.16 was approved by the national Medical Ethics Committee (CMO Arnhem‐Nijmegen) and was conducted in accordance with the principles of the Declaration of Helsinki. The participant gave informed consent before participation in this study. Subjects were allowed to leave this study at any time without any consequences.

### 2.3 Study design and treatment

**FIGURE 2:**
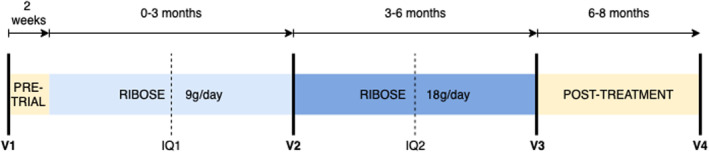
Overview of the study set‐up. With V1–V4 being visit one to four at which the participant visited the Radboud UMC outpatient clinic for measurements of our main outcome parameters. Intermediate Questionnaires (IQ1 and IQ2) consisted of three questionnaires and a 3‐day dietary intake journal.

Participant visited the Radboud University Medical Centre Muscle Centre outpatient clinic at 4 study days. Urine and blood samples were collected, and clinical assessment was performed by the same physical therapist for all participants (see below). Daily physical activity was measured using the GENEActiv Original accelerometer (ActivInsights) for 8 days following each visit, of which data of 7 full days were extracted for activity analysis. To assess variations in caloric intake, carbohydrates, proteins, and fats, the participant completed a 3‐day dietary intake journal during every visit (Figure 2: V1, V2, V3, and V4) and at two intermittent moments on fixed days (Figure 2: IQ1 and IQ2; sundays, mondays, and tuesdays). At these six time points (V1, V2, V3, V4, IQ1, IQ2) the participant was also asked to complete questionnaires on quality of life, pain, and level of physical activity, described in more detail below.

The total study period was 8 months of which the first 6 months were with ribose supplementation. Two weeks after visit 1, participant started the first period of supplementation with a daily dietary oral intake of ribose of 9 g in three doses per day for the first 3 months followed by 18 g per day for the last 3 months also divided in three doses per day (Bioenergy Life Science, Minnesota). After 6 months of supplementation, participant was followed for another 2 months without ribose intake (Figure 2). Therapy compliance was calculated as the percentage of ribose used by weighing the amount of remaining ribose after each visit compared to what was provided for the time period. Ribose has been used safely in humans for up to 60 g per day.^14^ In this study, ribose was given in a fed‐state, since supplementation with dietary ribose in healthy volunteers at a dose of 1 g/kg body weight given after overnight fasting resulted in transient hypoglycemia.^15^ The supplement was given in a powder form that needed to be dissolved in water before ingestion.

### 2.4 Clinical outcome measures

#### 2.4.1 Muscle strength assessment

Muscle strength was measured in 17 muscle groups using the Medical Research Council (MRC) score following standard protocol ranging from 0 to 5, with 0 being no contraction at all and 5 corresponding with normal muscle strength.^16^ MRC scores were measured bilateral for the following muscle groups: shoulder abduction (SA), shoulder forward flexion (SFF), elbow flexors (EF), elbow extensors (EE), wrist extensors (WE), wrist flexors (WF), handgrip (HG), pinchgrip (PG), hip extension (HE), hip flexion (HF), knee extension (KE), knee flexion (KF), ankle dorsiflexion (AD), ankle plantarflexion (AP), neck extension (NE), neck flexion (NF), and serratus anterior muscle (SEA). The maximum total MRC score was 160. HG was also measured using a JAMAR hand grip dynamometer.

Quantitative assessment of isometric muscle strength was performed for 11 muscle groups, using the MicroFET 2 hand‐held dynamometer according to standard protocol (Hogan Health Industries, Inc., West Jordan, UT, USA). The following muscle groups were tested bilateral: SA, EF, EE, HE, HF, KE, KF, NE, NF, SEA, and HG. All measurements were done in triplicate and means were calculated.

#### 2.4.2 Functional assessment

Domain 1 of the Motor Function Measure (MFM‐1), testing standing positions and transfers, was measured in which 13 items were scored on a scale from 0 to 3. MFM‐1 results are shown as a percentage of the maximum score, with 100% being equal to maximum muscle function.^17^ The other MFM domains were not assessed since they were not considered relevant for this disease. In order to test the participant's muscle strength endurance, the 45‐seconds Sit To Stand Test (45‐STST: participant had to stand up from a chair, without support of his hands, as many times as possible during 45 s) and a 12‐Minute Walking Test (12MWT) were performed.^18^ The participant's mobility was assessed by performing the Timed Up and Go Test (TUGT), measuring the time it takes to stand up from a chair, walk 3 m at a normal pace, return to the chair and sit down.^19^, ^20^ Measurements were done in duplicate and mean TUGT was calculated.

#### 2.4.3 Accelerometry

Accelerometry was used to measure total activity (counts/day), average activity (counts/s), and the percentages of a day (24 h) the participant spend sedentary, lightly, moderately, and vigorously active.^21^, ^22^

#### 2.4.4 Patient reported outcome measures

Participant completed three questionnaires at six time points: (a) The Frenchay Activities Index (FAI), a questionnaire that focuses on the participant's physical activity levels, as well as social functioning. The items in this questionnaire are scored on a scale from 0 to 3, with a maximum score of 45 points indicating high levels of physical and social activity.^23^ (b) The Checklist Individual Strength (CIS), which uses 20 items to measure subjective fatigue and behavioral aspects on a seven‐point scale, with a maximum score of 140 indicating a patient experiences problematic fatigue.^24^ (c) The McGill Pain Questionnaire (MPQ), a questionnaire in which the location, intensity, and characteristics of pain were assessed. The MPQ uses 20 sets of three to four descriptive adjectives ordered in intensity, from which the participant had to choose the words that describe his current pain. These choices were then converted into two main measures: the Total Number of Words Chosen (NWC‐T) and the Total Pain Rating Index (PRI‐T), which is the sum of the intensities of the chosen words. The maximum score for the NWC‐T is 20, and the maximum PRI‐T score is 63. The higher these scores, the more pain the participant experienced. In the MPQ, pain scores were also reported using a Visual Analogue Scale (VAS) ranging from 0 to 10.^25^

### 2.5 Biochemical measurements

#### 2.5.1 Diagnostic evaluations

Measured biochemical parameters included blood urate and glucose levels (fed state), since ribose is known to possibly induce mild hypoglycemia and hyperuricemia.^15^, ^26^ Furthermore, blood creatine kinase (CK), a known biomarker for muscle damage was measured.

### 2.6 Metabolomics liquid chromatography‐mass spectrometry (LC–MS) measurements

Metabolite levels were determined in triplicates by LC–MS according to the polar metabolite extraction protocol used by van Tol et al. with slight adjustments.^9^ In short, blood was drawn in heparin tubes and immediately centrifuged to obtain erythrocyte fractions. Of this fraction 100 μL was incubated for 5 min with 1:4 ratio ice‐cold (−20°C) methanol:acetonitrile (AcN) 1:1 (v:v) and centrifuged twice at 16 000 × *g* for 3 minutes at 4°C and after each time supernatant was transferred to a new eppendorf tube. Supernatants were centrifuged and dried with a centrifugal vacuum concentrator (SpeedVac, Thermo Fisher Scientific) and stored at −80°C before LC–MS measurements. The LC–MS method used was described in van Scherpenzeel et al.^27^ Separation of nucleotide sugars was achieved using ion pair‐reverse phase chromatography. Using an ultra‐high performance liquid chromatography (UPLC) system (Agilent 1290 Infinity), 10 μL of erythrocyte extract was injected onto a HSS T3 column (Waters, 2.1 × 150 mm i.d., 1.8‐μm particle size) which was set to 25°C. Chromatography was performed with 350 μL/min flow rate, 35 min total run time, and a gradient from 0% to 10% mobile phase B; 10.0 min: 0% B; 15 min: 4% B; 25 min: 4% B; 26 min: 10% B; 27 min: 10% B; 28 min: 0% B. Mobile phase A consisted of 20 mM triethylammonium acetate acid (TEAA, Merck) in MiliQ, mobile phase B consisted of 50% ACN/MiliQ (v/v). UPLC was coupled to an Agilent 6490 QqQ LC/MS with high‐flow iFunnel ionization source. Acquisition parameters were set with the following values: 3500 V capillary voltage and 2000 V nozzle voltage, drying gas flow of 15 L/min at 200°C, sheath gas (nitrogen) flow of 12 L/min at 200°C, nebulizer gas flow set at 20 psi, MS operating pressure of 5 × 10^−5^ Torr, Q1 and Q3 were set to unit resolution (0.7 full width at half maximum [FWHM]), and 10 ms dwell time. Fragmentor voltage was 380 V at default and cell accelerator potential was 4 V for all multiple reaction monitoring (MRM) ion pairs. A minimum of three transitions were determined for each compound. Manual interpretation and verification of peaks integration was performed using Skyline (version 21.2.0.425) using standards and retention time. Absolute intensities were exported and relative normalization was performed within samples.

### 2.7 Data collection and analysis

Data collection was performed using a printed case report form (CRF) and data was pseudonymized using a personal identification number for each participant. Printed and digital data were stored at the Radboud UMC, Department of Neurology.

An in‐house MATLAB script was used to calculate total activity, average activity, and the percentages of sedentary, light, moderate, and vigorous activity from the extracted accelerometer data. Descriptive statistical analyses for muscle strength, functional assessments, and patient reported outcome measures were performed using IBM SPSS Statistics 25.0.

For data interpretation of LC–MS data, individual nucleotide sugars were normalized using total peak area to obtain their relative abundance. Differential analysis and visualization was performed using GraphPad Prism 9.

## 3 RESULTS

### 3.1 Patients and therapy

We recruited patients among the members of the national patient organization Spierziekten Nederland. The two patients who expressed their interest at an annual meeting were invited. One other patient with ISPD mutations was invited, but he declined the invitation because of comorbidity (stroke).

Two patients were included with LGMD2I (OMIM #607155) due to a homozygous mutation in the *FKRP* gene. However, one patient was excluded for analyses due to noncompliance of ribose supplementation. Patient characteristics at baseline are shown in Table 1. The patient showed mild limb girdle muscle weakness with elevated CK without central nervous system abnormalities. He has a history of cardiomyopathy with an ejection fraction of 28% in 2017, for which he was prescribed carvedilol (orally 12.5 mg three times per day), eplerenone (orally 12.5 mg every day), lisinopril (orally 10 mg every day), and he received an implantable cardioverter defibrillator (ICD) in 2018. Lisinopril was increased to 10 mg twice per day during this study, while other medication remained unchanged.

**TABLE 1:** Patient characteristics at baseline.

Sex	Male
Age (years)	35
Age at diagnosis	18
Mutation	c.826C>A; p.Leu276Ile homozygous
Height (cm)	184.8
Weight (kg)	91.1
BMI (kg/m^2^)	26.9

No safety issues arose during ribose supplementation. Body weight and body mass index (BMI) stayed stable throughout this study (Table 2).

**TABLE 2:** Dietary intake and BMI.

	V1	IQ1	V2	IQ2	V3	V4
Weight (kg)	91.1	N/A	91.0	N/A	92.5	92.0
BMI (kg/m^2^)	26.9	N/A	26.2	N/A	26.9	26.3

Abbreviations: IQ1, intermediate questionnaires 1; IQ2, intermediate questionnaires 2; N/A, not applicable; V1, visit 1; V2, visit 2; V3, visit 3; V4, visit 4.

### 3.2 Therapy compliance

For the patient included in this study therapy compliance was 99% for the dose of 9 g/day and 98% for 18 g/day.

### 3.3 Clinical outcome measures

**FIGURE 3:**
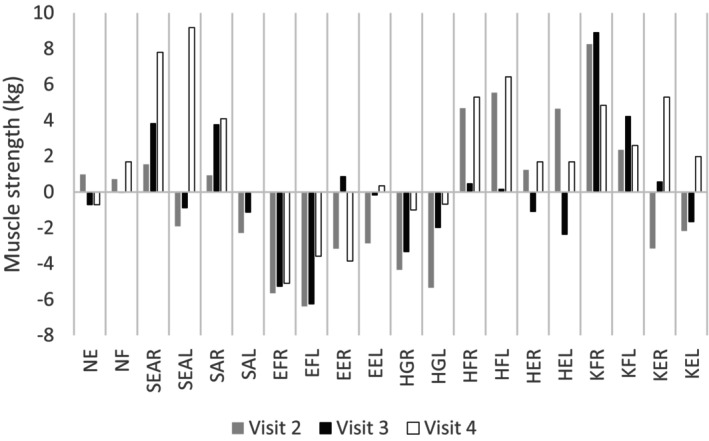
Strength measurements using the MicroFET hand‐held dynamometer. Results are shown as change in muscle force when compared to baseline (visit 1). NE = neck extension; NF = neck flexion; SEAR = serratus anterior right; SEAL = serratus anterior left; SAR = shoulder abduction right; SAL = shoulder abduction left; EFR = elbow flexion right; EFL = elbow flexion left; EER = elbow extensors right; EEL = elbow extensors left; HGR = handgrip right; HGL = handgrip left; HFR = hip flexion right; HFL = hip flexion left; HER = hip extension right; HEL = hip extension left; KFR = knee flexion right; KFL = knee flexion left; KER = knee extension right; KEL = knee extension left.

The results of the clinical outcome measures are shown in Table 3 and Figure 3. Comparing visit 2 and 3 to visit 1, the patient showed an improvement in total MRC score, however after supplementation (visit 4), MRC scores increased even more. Quantitative strength measurements using hand‐held dynamometry showed no clear improvement or deterioration when comparing muscle strength. MFM‐1 scores improved and at visit 1 and 2 the patient was unable to perform the 45‐STST, whereas in visit 3 he could stand up 10 times during 45 s. 12‐MWT distance remained stable. Total and average daily activity, measured by accelerometry, slightly increased every visit, however the percentages of time spend sedentary, lightly, moderately, and vigorously active remained stable indicating that physical activity levels remained stable during the course of this study.

**TABLE 3:** Clinical outcome measures.

	Patient			
	V1	V2	V3	V4
MRC total score (0–160)	149	157	153	157
MFM‐1 (0–100%)	79	82	90	90
TUGT, mean (s)	4.68	6.93	7.12	6.69
45‐STST	0	0	10	9
Distance 12‐MWT (m)	961	977	978	939
Accelerometry				
Total activity (counts/day)	257 460	283 680	288 250	350 320
Average activity (counts/s)	2.98	3.28	3.34	4.05
Sedentary (average % per day)	90.9	91.2	88.9	89.3
Light (average % per day)	5.7	5.9	7.0	6.7
Moderate (average % per day)	3.1	2.6	3.6	3.6
Vigorous (average % per day)	0.3	0.3	0.5	0.4

Abbreviations: 12‐MWT, 12 Minute Walking Test; 45‐STST, 45 seconds Sit To Stand Test; MFM‐1, Motor Function Measure domain 1; MRC, Medical Research Council; TUGT, Timed Up and Go Test.

Patient reported outcome measures are shown in Table 4. The patient did not report any improvement when using 9 g of ribose per day. Whereas he reported a subjective improvement in muscle strength and endurance, less fatigue, and less pain when using 18 g of ribose per day. However, when comparing questionnaire scores of visit 3 to visit 1, he reported more fatigue (shown by a higher CIS total score) and only slight improvements were seen in FAI, MPQ, and VAS scores.

**TABLE 4:** Patient reported outcome measures derived from questionnaires.

	V1	IQ1	V2	IQ2	V3	V4
FAI (0–45)	41	41	41	40	42	38
CIS total score (0–140)	45	53	39	47	57	57
MPQ, NWC‐T (0–20)	11	12	12	15	9	14
MPQ, PRI‐T (0–63)	17	21	16	28	12	23
VAS (0–10)	5.8	2.6	2.9	2.6	4.5	4.5

Abbreviations: CIS, Checklist Individual Strength; FAI, Frenchay Activity Index; MPQ‐DLV, McGill Pain Questionnaire; NWC‐T, Number of Words Chosen Total; PRI‐T, Pain Rating Index Total; VAS, Visual Analogue Scale.

### 3.4 Biochemical measurements

#### 3.4.1 Diagnostic lab levels

**FIGURE 4:**
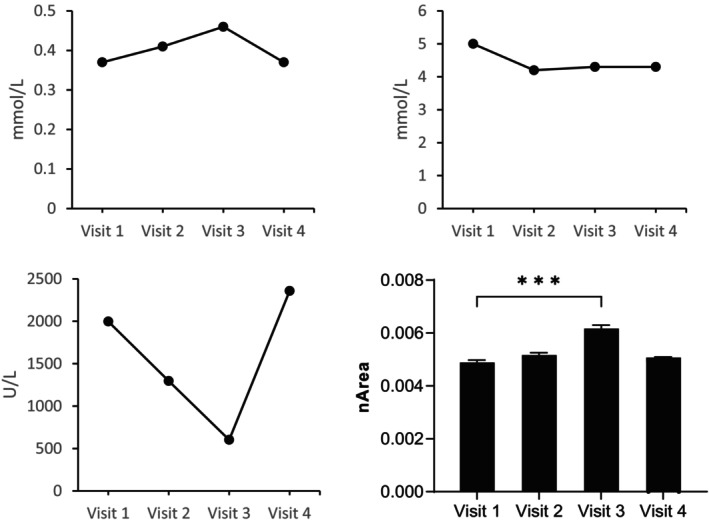
Biochemical levels of urate, glucose, and creatine kinase in blood. (A) Blood urate levels. (B) Blood glucose levels (fed state). (C) Blood creatine kinase levels. (D) Mean normalized area of CDP‐ribitol levels in erythrocytes. CDP, cytidine diphosphate.

Blood urate and glucose levels remained stable and within reference ranges (Figure 4A, B and Table S1) during all four visits. Blood CK levels decreased from 1997 U/L at baseline to 1297 U/L at visit 2, which was then decreased by more than 50% at visit 3 (602 U/L) and increased back to 2358 U/L at visit 4, see Figure 4C. Interestingly, the patient has a history of cardiomyopathy. During this study an increase of his ejection fraction from 32% in 2020 to 38% in 2021 was recorded at his semi‐annual check‐up at the cardiologist. This check‐up was scheduled during the period of 18 g/day ribose supplementation.

#### 3.4.2 Metabolomics LC–MS measurements

CDP‐ribitol levels were measured in erythrocytes in order to study a possible biochemical effect of ribose supplementation. A small and dose‐dependent increase in CDP‐ribitol levels was observed from visits 1 to 3, while at visit 4, CDP‐ribitol levels were more comparable to baseline levels (Figure 4D). An overview of all results from the LC–MS measurements is shown in Appendix B1.

## 4 DISCUSSION

In this study we report the safety and effects of oral ribose supplementation during 6 months in a patient with LGMD type 2I (LGMD2I) due to a homozygous *FKRP* mutation. Ribose was chosen, since this supplement has proven to be safe, is commercially available and unlike ribitol does not require clinical trials.^11^, ^12^, ^13^ This study showed that ribose can be used safely in a dose of both 9 g and 18 g per day during periods of 3 months in a patient with genetically confirmed dystroglycanopathy due to an *FKRP* mutation. Four important findings emerged. First, the patient reported a subjective improvement of muscle strength, fatigue, and pain. This resulted in a subjective improvement in his functional tasks in his day‐to‐day life as well. Second, while he was unable to stand up during the 45‐STST at baseline, after supplementation with 18 g of ribose he was able to stand up 10 times during 45 s. Third, blood CK levels decreased by 70% during supplementation with 18 g of ribose compared to baseline. And fourth, erythrocyte CDP‐ribitol levels slightly increased in a dose‐dependent manner, suggesting a correlation with improved glycosylation of αDG. We will discuss these findings below.

Two of the clinical observations above (improved function on 45‐STST and subjective improvement of symptoms) might point to a mild therapeutic effect of ribose. However, no clear improvement in clinical and patient reported outcome measures was observed. The results of the strength measurements (MRC scores and HHD) were inconclusive and showed a high level of variability, questioning the validity of these measurements in a single patient with a disease of varying intensity. Furthermore, only small improvements in patient reported outcome measures were seen, however patients are subject to variability in experienced symptoms daily.

Although this study focused on a possible effect of ribose on skeletal muscle, the heart may also benefit from ribose treatment. This has been supported by literature on the positive effect of ribose on myocardial function in congestive heart failure and ischemic cardiovascular diseases.^28^, ^29^, ^30^ An increase of the patient's ejection fraction from 32% in 2020 to 38% in 2021 was reported by his cardiologist during the period of 18 g/day ribose supplementation. After completion of the study he continued using 12 g of ribose per day, skipping the dose taken with breakfast as he usually skips breakfast. At his following check‐up his cardiologist reported an ejection fraction of 32%. This could suggest that a minimum level of ribose is required for heart related effects, which requires further study. However, this does indicate the importance of including cardiologic measurements, such as ejection fraction, in future studies in dystroglycanopathies.

One of the observed metabolic effects was a decrease in CK levels during the months of supplementation. Increased levels of CK are nonspecific markers for muscle integrity and often increased during muscle stress and injury.^31^ The patient did not report an increase in VAS score, however his activity levels increased according to the accelerometry. CK levels decreased more with higher doses of supplementation and restored to baseline level after the period without supplementation, indicating a possible effect of ribose on lowering CK levels and its possible effect on functional outcome measures.

A second metabolic parameter was the CDP‐ribitol level measured in erythrocytes, which is a minimally invasive way of gaining information on metabolic level which could give an indication of effect as it is vital for glycosylation of αDG (Figure 1). We showed an increase in CDP‐ribitol levels during supplementation. However, measuring CDP‐ribitol levels in muscle tissue derived from a muscle biopsy would result in a better estimation of the effects of the supplement on glycosylation levels at the site of action (muscle tissue). This is particularly interesting as studies have shown association between functional glycosylation of αDG and muscle regeneration.^32^ Using tissue samples, localization of FKRP can also be studied as there are indications that mislocalization could possibly alter its function in muscle cells.^33^ However, in this case study we opted not to have a muscle biopsy due to the burden it has on the patients.^34^

In accordance with previous studies, ribose has been safely administered to our patient.^11^, ^35^ Previous studies investigating the use of ribose as a dietary supplement reported transient hypoglycemia and hyperuricemia.^15^, ^26^ This was not observed in our patient, and we therefore recommend ribose to be used in a fed‐state. Due to the frequent dosage, it should be considered whether other forms of ribose are possible for administration, for example, oral tablets or capsules which are easier to ingest and would not need to be dissolved in water.

In order to specifically test the effects of ribose supplementation, we aimed to control the caloric intake by using dietary control from a 3‐day dietary intake journal and monitoring activity by accelerometry analyses. Interestingly however, a decrease in caloric intake was seen over the course of this study, whereas body weight remained stable, indicating possible inconsistency in filling out the dietary intake journals. We therefore recommend using body weight and bioimpedance analysis (BIA) measurements as a control measure for dietary intake.

Previous research focused on the potential effect of ribose on patients with an *ISPD* mutation, since ISPD is the enzyme that produces CDP‐ribitol.^9^ However, the patient included in the current study had an *FKRP* mutation which highlights the importance of further investigating the effect of ribose supplementation on other genetic defects involved in this complex pathway.

In addition, this study is limited by the number of patients participating. One improvement could be to expand the study in the future to a multicenter setting to increase recruitment. Furthermore, long‐term effects of ribose remain to be elucidated. Perhaps the amount of ribose can be adjusted to a higher dose. Another possibility is to add another supplement to enhance its effect. Recently, it was shown that NAD+ enhanced the rescue with ribitol and ribose on induced pluripotent stem cell (iPSC) derived FKRP‐mutant myotubes.^36^ As NAD+ is commercially available this could be subject to future trials. Furthermore the patient was only mildly affected. Hence, our data are not representative for the wide clinical spectrum of LGMD2I. Finally, we had not included muscle imaging outcome measures (muscle ultrasound or muscle MRI), since we expected that this would not show significant changes during the treatment period. In a future trial with a longer duration, this might be a valuable biomarker.

In short, this study shows that the use of ribose is safe, has the potential to lower blood CK levels and increase CDP‐ribitol levels in erythrocytes. Although the patient reported subjective improvements on muscle strength, fatigue, and pain, this was not seen in clinical and patient‐reported outcome measures. Dietary ribose therapy has the potential to function as a cheap and safe therapy in dystroglycanopathy patients. Therefore, future research in a larger cohort is needed to elucidate the potentially beneficial effects of this supplement in dystroglycanopathy patients with an *FKRP, FKTN*, or *ISPD* mutation.

## CONFLICT OF INTEREST STATEMENT

The authors declare no conflicts of interest.

## ETHICS STATEMENT

This study with number NL58620.091.16 was approved by the national Medical Ethics Committee (CMO Arnhem‐Nijmegen) and was conducted in accordance with the principles of the Declaration of Helsinki. The participants gave informed consent before participation in this study. Subjects were allowed to leave this study at any time without any consequences.

## INFORMED CONSENT

This study with number NL58620.091.16 was approved by the national Medical Ethics Committee (CMO Arnhem‐Nijmegen) and was conducted in accordance with the principles of the Declaration of Helsinki as well as institutional and national legislation. The participant gave informed consent for sharing of patient information and to use body fluid samples for research purposes before participation in this study. Subject was allowed to leave this study at any time without any consequences.

## Supporting information


**Data S1.** Supporting Information.

## Data Availability

Data presented in this study are available upon reasonable request from the corresponding author.
