# Supplementary material for: Oral ribose supplementation in dystroglycanopathy: A single case study
Source: JIMD Rep. 2024 Mar 4;65(3):171–81. doi: 10.1002/jmd2.12394 (PMC11078721; doi:10.1002/jmd2.12394)
Supplement: Supplementary file 1 — Data S1. Supporting Information. [file JMD2-65-171-s001.docx]

# Appendix

## Appendix A – Reference ranges

| Biochemical parameter | Reference range, men |
| --- | --- |
| Blood urate (mmol/L) | 0.20 – 0.42 |
| Blood creatine kinase (U/L) | <200 |
| Blood glucose, fed state (mmol/L) | 3.5 – 7.8 |

## Appendix B1 – LC-MS measurements patient 1

Appendix B1 shows an overview of the analyzed nucleotide sugars in patient 1 in relative abundances (%). In grey CDP-ribitol.

|  | ADP-D-ribose | AMP | CDP-Ribitol | CMP-N-acetyl-neuraminic acid | CMP-N-glycoloyl-neuraminic acid | Cytidine monophosphate | GDP-Fucose | GDP-Mannose | GMP | UDP-Arabinose | UDP-D-galactose | UDP-Glucose | UDP-glucuronic acid | UDP-Mannose | UDP-N-acetyl-hexosamine | UDP-xylose | Uridine 5'-monophosphate |
| --- | --- | --- | --- | --- | --- | --- | --- | --- | --- | --- | --- | --- | --- | --- | --- | --- | --- |
| Patient 1 erythrocytes (visit 1) average | 0.022267 | 0.090266 | 0.004888 | 0.003044 | 0.000452 | 5.13E-05 | 0.017685 | 0.0131 | 0.00021 | 0.000671 | 0.09732 | 0.360185 | 0.013506 | 0.011713 | 0.368335 | 0.000602 | 0.000592 |
| Standard deviation | 0.001408 | 0.002084 | 0.000157 | 5.3E-05 | 0.000102 | 4.34E-06 | 0.000868 | 0.000894 | 3.86E-05 | 1.96E-05 | 0.000745 | 0.003324 | 0.000942 | 0.000264 | 0.005256 | 5.77E-05 | 0.0004 |
| Patient 1 erythrocytes (visit 2) average | 0.016081 | 0.140881 | 0.005164 | 0.005072 | 0.000406 | 0.000135 | 0.016603 | 0.013533 | 0.000456 | 0.000681 | 0.092104 | 0.34937 | 0.015157 | 0.011589 | 0.33483 | 0.000624 | 0.002479 |
| Standard deviation | 0.003203 | 0.003471 | 0.000162 | 0.00014 | 0.000142 | 0.000116 | 0.000496 | 0.00035 | 3.74E-05 | 2.45E-05 | 0.002087 | 0.003077 | 0.000151 | 0.000129 | 0.002444 | 5.6E-05 | 0.002014 |
| Patient 1 erythrocytes (visit 3) average | 0.016776 | 0.086693 | 0.006168 | 0.005158 | 0.000467 | 8.66E-05 | 0.018018 | 0.014014 | 0.000277 | 0.00073 | 0.103663 | 0.379564 | 0.017226 | 0.012006 | 0.342145 | 0.000604 | 0.002571 |
| Standard deviation | 0.001228 | 0.004756 | 0.000223 | 6.59E-05 | 0.000133 | 3.24E-05 | 0.000678 | 0.000838 | 4.86E-05 | 2.92E-05 | 0.001923 | 0.003301 | 0.000726 | 0.000345 | 0.00195 | 1.64E-05 | 0.001072 |
| Patient 1 erythrocytes (visit 4) average | 0.01505 | 0.086831 | 0.00507 | 0.00351 | 0.000346 | 5.95E-05 | 0.019212 | 0.015648 | 0.000343 | 0.000674 | 0.100875 | 0.375599 | 0.015685 | 0.012473 | 0.352002 | 0.000599 | 0.001091 |
| Standard deviation | 0.000238 | 0.002139 | 5.32E-05 | 4.46E-05 | 0.000154 | 1.58E-05 | 0.000397 | 0.000434 | 3.04E-05 | 3.3E-05 | 0.000877 | 0.004639 | 0.000646 | 0.000573 | 0.003554 | 5.53E-05 | 0.000408 |
|  |  |  |  |  |  |  |  |  |  |  |  |  |  |  |  |  |  |
